# Revealing Differential RNA Editing Specificity of Human ADAR1 and ADAR2 in *Schizosaccharomyces pombe*

**DOI:** 10.3390/genes15070898

**Published:** 2024-07-09

**Authors:** Niubing Zhang, Ping Chen, Zilin Cui, Xiaojuan Zhou, Chenhui Hao, Bingran Xie, Pei Hao, Bang-Ce Ye, Xuan Li, Xinyun Jing

**Affiliations:** 1 State Key Laboratory of Bioreactor Engineering, East China University of Science and Technology, Shanghai 200237, China; 2 Key Laboratory of Synthetic Biology, Key Laboratory of Plant Design, CAS Center for Excellence in Molecular Plant Sciences, Chinese Academy of Sciences, Shanghai 200032, China; 3 University of Chinese Academy of Sciences, Beijing 100039, China; 4 State Key Laboratory of Crop Stress Adaptation and Improvement, School of Life Sciences, Henan University, Kaifeng 475004, China; 5 Key Laboratory of Molecular Virology and Immunology, Institut Pasteur of Shanghai, Chinese Academy of Sciences, Shanghai 200031, China

**Keywords:** A-to-I RNA editing, ADAR, editing specificity, recoding

## Abstract

Adenosine-to-inosine (A-to-I) RNA editing is an important post-transcriptional modification mediated by the adenosine deaminases acting on RNA (ADAR) family of enzymes, expanding the transcriptome by altering selected nucleotides A to I in RNA molecules. Recently, A-to-I editing has been explored for correcting disease-causing mutations in RNA using therapeutic guide oligonucleotides to direct ADAR editing at specific sites. Humans have two active ADARs whose preferences and specificities are not well understood. To investigate their substrate specificity, we introduced hADAR1 and hADAR2, respectively, into *Schizosaccharomyces pombe* (*S. pombe*), which lacks endogenous ADARs, and evaluated their editing activities in vivo. Using transcriptome sequencing of *S. pombe* cultured at optimal growth temperature (30 °C), we identified 483 A-to-I high-confident editing sites for hADAR1 and 404 for hADAR2, compared with the non-editing wild-type control strain. However, these sites were mostly divergent between hADAR1 and hADAR2-expressing strains, sharing 33 common sites that are less than 9% for each strain. Their differential specificity for substrates was attributed to their differential preference for neighboring sequences of editing sites. We found that at the -3-position relative to the editing site, hADAR1 exhibits a tendency toward T, whereas hADAR2 leans toward A. Additionally, when varying the growth temperature for hADAR1- and hADAR2-expressing strains, we observed increased editing sites for them at both 20 and 35 °C, compared with them growing at 30 °C. However, we did not observe a significant shift in hADAR1 and hADAR2’s preference for neighboring sequences across three temperatures. The vast changes in RNA editing sites at lower and higher temperatures were also observed for hADAR2 previously in budding yeast, which was likely due to the influence of RNA folding at these different temperatures, among many other factors. We noticed examples of longer lengths of dsRNA around the editing sites that induced editing at 20 or 35 °C but were absent at the other two temperature conditions. We found genes’ functions can be greatly affected by editing of their transcripts, for which over 50% of RNA editing sites for both hADAR1 and hADAR2 in *S. pombe* were in coding sequences (CDS), with more than 60% of them resulting in amino acid changes in protein products. This study revealed the extensive differences in substrate selectivity between the two active human ADARS, i.e., ADAR1 and ADAR2, and provided novel insight when utilizing the two different enzymes for in vivo treatment of human genetic diseases using the RNA editing approach.

## 1. Introduction

Adenosine-to-inosine (A-to-I) RNA editing is a posttranscriptional modification performed by adenosine deaminases acting on RNA (ADAR), which can bind to double-stranded RNA (dsRNA) and deaminate adenosine to inosine [1]. This process predominantly occurs on mRNA, microRNA, and repetitive elements such as inverted *Alu*s and short interspersed nuclear elements (SINEs) [2]. Upon translation, inosine is interpreted by the ribosomal machinery as guanosine, so RNA A-to-I editing can result in protein variants with altered amino acid composition, termed “recoding”. Additionally, RNA A-to-I editing can change alternative splicing and generate both gain and loss of microRNA-binding to target mRNA [3,4,5]. RNA editing sites in repetitive elements, altering dsRNA structures, have the purpose of escaping recognition as non-self by the host immune system [6].

ADARs, highly conserved across all metazoans, are not observed in bacteria, fungi, or plants. Mammals express three main ADAR proteins: ADAR1, ADAR2, and ADAR3 [7]. In contrast, *Drosophila* possesses only one type of ADAR, similar in sequence and function to mammalian ADAR2 [8]. Cephalopods possess both ADAR1 and ADAR2 [9]. All ADAR proteins share common features, including at least one N-terminal double-stranded RNA-binding domain (dsRBD) and one C-terminal deaminase domain. ADAR2, for instance, has two dsRNA-binding domains and a deaminase domain, predominantly located in the nucleus of mammalian cells [10]. ADAR1 exists in two isoforms, p110 and p150 [11]. The shorter ADAR1 p110 isoforms, located in the nucleus, include a Z-DNA binding domain, three dsRNA binding domains, and a deaminase domain, predominantly found in the nucleus. Conversely, the larger isoform p150 harbors an additional Z-DNA binding domain and is located in the cytoplasm. ADAR3 contains two dsRNA binding domains and a deaminase domain but lacks the RNA A-to-I editing activity [12].

ADAR1 and ADAR2 exhibit overlapping RNA editing sites [13], with 262 shared sites in humans [14] and 2849 in mice [15]. Across human [16,17], mouse [15,18], *Drosophila* [19], *Xenopus* [20,21], and octopus ADARs [22], a common preference for editing adenosine preceded by uracil and a downstream bias towards guanine is observed. However, different ADAR family members may target distinct sites. In human cells, overexpression of hADAR1 and hADAR2 revealed 9352 and 1403 associated editing sites, respectively, which account for 20% and 2.8% of all editing sites in repetitive regions [14]. ADAR1 edits millions of A-to-I sites within repeats, such as human *Alu* elements, whereas ADAR2 predominantly edits exonic regions, especially CDS in mRNAs [14]. In mice, ADAR1 and ADAR2 knockout models demonstrated a similar number of editing sites with comparable genome-wide and repeat region distributions. Nonetheless, subtle preferences in neighboring bases were noted; mouse ADAR1 favors thymine at the −2 and adenine at the +2 positions relative to the target adenosine, while ADAR2 shows a preference for cytosine at −2 and guanine at +2 [15]. Furthermore, human ADAR2 is characterized by its ability to induce editing at the −26 position in broken double-stranded structures, contrasting with ADAR1, which targets the −35 position [23]. This variation is attributed to differences in the number and composition of RNA-binding domain (RBD) amino acids present in hADAR1 and hADAR2.

The extent of RNA A-to-I editing at a specific site is influenced by both the sequence context and the dsRNA structure, including factors like length and interruptions in base-pairing caused by mismatches, bulges, or loops [24]. The neighbor preferences derive from the catalytic domain [21], while the presence of dsRBDs in ADARs explains the necessity for a double-stranded secondary structure of specific length in known RNA editing substrates [16]. The dsRBDs are involved in dsRNA substrate recognition and RNA binding and play a role in ADAR selectivity via both sequence-specific and non-specific mechanisms [25].

While mammalian ADAR enzymes display distinct preferences for genomic regions, their substrates and related functions are poorly defined. In vitro studies using an approximately 800-bp dsRNA substrate have revealed the 5′ nearest-neighbor preference of U > A > C > G and the 3′ nearest-neighbor preference of G > C~A > U for human ADAR1 (hADAR1) and G > C > U~A for human ADAR2 (hADAR2) [21]. However, the significant overlap in the editing capabilities of ADAR1 and ADAR2 in vivo in mammalian cells [14] complicates the determination of each ADAR’s contribution to RNA editing in vivo. Three approaches have been employed to address this issue. The first method involves culturing HeLa cell lines with RNAi-mediated knockdown of either ADAR1 or ADAR2, followed by the analysis of editing sites specific to each enzyme [26]. The second method involves inducing the expression of ADAR1 p150 through IFN-stimulation in HEK293T cell lines, or overexpressing ADAR2, and comparing changes in editing ratios to ascertain the sites targeted by each enzyme [14]. The third approach investigates the editing specificity and tissue distribution of ADAR1 and ADAR2 using *Adar1*−/− or *Adar2*−/− mice. Research employing *Adar1*−/− or *Adar2*−/− mutant mice has achieved significant progress [14]. However, these research methods still have some limitations. For instance, the first and second methods rely solely on correlational analysis between changes in ADAR enzyme expression levels and changes in editing levels at specific sites to determine attribution to ADAR1 or ADAR2. Since complete elimination of either enzyme has not been achieved, residual enzyme activity may affect site-specific editing analysis for two enzymes. Additionally, potential *trans* regulators in animal cells, such as ADAR3, AIMP2, and others, may impact ADAR enzyme activity, rendering the study of ADAR’s specificity and efficiency in cell lines and *Adar1*−/− or *Adar2*−/− tissues less reflective of the true situation. To overcome these limitations, we wondered whether we could use yeast cells that lack the A-to-I RNA editing machinery to create a platform with a clean background for studying the specificity of hADAR1 and hADAR1, the regulation of temperature on RNA editing efficiency and specificity*,* and the impact of RNA editing on gene functions.

In this study, we initially introduced the RNA editing machinery into *S. pombe* and confirmed its editing activity in vivo. Subsequently, we conducted RNA-seq analysis on *S. pombe* strains containing the RNA editing machinery and control strains. We identified 483 to 1656 RNA A-to-I editing sites for hADAR1 and 404 to 5997 sites for hADAR2 across different temperatures. Upon comparing editing sites between hADAR1 and hADAR2, we observed distinct RNA editing sites for hADAR1 and hADAR2, which is related to the different preferences of neighboring sequences of the editing sites between the two enzymes. By comparing the editing sites of a specific enzyme across three temperatures, we found that both hADAR1 and hADAR2 exhibited varied editing specificity across different temperatures, although their preference for neighboring sequences did not exhibit a significant shift across temperatures. Additionally, we observed that genes’ functions can be greatly affected by the editing of their transcripts, in which over 50% of RNA editing sites were located in exons and more than 60% of editing sites resulted in protein recoding.

## 2. Materials and Methods

### 2.1. Construction of hADAR1 and hADAR2 Expression Vectors

To generate hADAR1 and hADAR2 expression vectors for yeast cells, we utilized an *Esherichia coli—S. pombe* shuttle plasmid vector pDUAL-HFF1 [27]. The plasmid pDUAL-HFF1 (RIKEN BioResource Research Center, Tsukuba, Japan) was digested with the *Nhe* I/*Bgl* II restriction enzymes (New England Biolabs, Ipswich, MA, USA), before it was assembled with the amplified hADAR1 or hADAR2 PCR fragments, respectively, producing plasmids pDUAL-HFF1-hADAR1 and pDUAL-HFF1-hADAR2. To facilitate chromosomal integration of hADAR expression constructs, both plasmids were digested with the *Not* I restriction enzyme (Thermo Scientific, Waltham, MA, USA) and treated with FastAP Thermosensitive Alkaline Phosphatase (Thermo Scientific, Waltham, MA, USA). Then, the linearized DNA was purified by agarose gel electrophoresis, preparing it for transformation into yeast.

### 2.2. S. pombe Strains and Transformation

Strains FY7652-hADAR1, FY7652-hADAR2, and FY7652-HFF1 originated from the *S. pombe* FY7652 (h-*leu1*-32 *ura4*-D18) strain (National BioResource Project, Osaka, Japan). Transformation of the yeast strains was performed using the Lithium Acetate/PEG/Heat shock method [28] with 500 ng of linear plasmid DNA. Following transformation, colonies were selected on EMM plates supplemented with 50 μg/mL uracil and then incubated at 30 °C for 3 days. Verification of correct integration of hADAR1, hADAR2, or empty constructs into the chromosomal DNA was conducted via PCR analysis using primer sets ADHterm-F and leu1-R [29].

### 2.3. Western Blotting

Strains FY7652-hADAR1, FY7652-hADAR2, and FY7652-HFF1 were grown in EMM supplemented with 50 μg/mL uracil until stationary phase. Subsequently, cells were harvested and lysed using lysis buffer. The resulting protein samples were mixed with 5 × SDS loading buffer (Yeasen, Shanghai, China) and subjected to boiling. Then, the samples were separated on 15% SDS-PAGE gels and transferred to a PVDF membrane. The membrane was then probed with either anti-ADAR1 mouse monoclonal antibody (1:500 dilution, Santa Cruz Biotechnology, Santa Cruz, CA, USA) or rabbit anti-ADAR2 polyclonal antibody (1:1000 dilution, ABClonal, Woburn, MA, USA), followed by HRP-conjugated IgG antibody (ABClonal, Woburn, MA, USA).

### 2.4. S. pombe Strains Cultivation

The strains FY7652-hADAR1, FY7652-hADAR2, and FY7652-HFF1 were initially plated in EMM supplemented with 50 μg/mL uracil, and grown at 30 °C for 3 days [30]. Colonies were then picked and employed to seed cultures of 3 mL of EMM supplemented with 50 μg/mL uracil at 30 °C, 220 rpm, and grown until mid-log phase. Harvested cells were inoculated in 20 mL of EMM supplemented with 50 μg/mL uracil, with an initial concentration (OD_600_) of 0.1. They were cultured at different temperatures (20, 30, and 35 °C) until mid-log phase, after which the cells were harvested for RNA extraction and sequencing.

### 2.5. Transcriptome Sequencing (mRNA-Seq)

mRNA-seq was performed in our previous report [31]. Briefly, total RNA was extracted from FY7652-hADAR1, FY7652-hADAR2, and FY7652-HFF1 strains using RNeasy Mini kit (Qiagen, Venlo, The Netherlands) following the manufacturer’s instructions. The integrity of RNA (RIN) was assessed using an Agilent 2100 Bioanalyzer (Agilent Technologies, Santa Clara, CA, USA). mRNA was purified from total RNA using poly-T oligo, and sequencing libraries were constructed using TruSeq RNA Library Preparation Kit (Illumina, San Diego, CA, USA). The libraries were sequenced on an Illumina Novaseq platform, and 150 bp PE reads were generated (Shanghai Biotechnology Corporation, Shanghai, China).

### 2.6. Pipeline for Identification of A-to-I RNA Editing Events

The pipeline for the identification of A-to-I RNA editing events was modified from that used in our previous work [31,32]. Firstly, the raw transcriptome sequencing data were processed to remove low-quality reads. Reads were initially trimmed from the 5′ end using Fastx_trimmer (version 0.0.13) with options -f11 -Q33. Replicate reads were then eliminated using FastUniq (version 1.1) [33], followed by the utilization of sickle (version 1.33, https://github.com/najoshi/sickle, accessed on 29 March 2019) to remove low-quality reads with options “-l 50 -n -q 30”. The resulting clean datasets were assessed using FastQC (version 0.11.8, http://www.bioinformatics.babraham.ac.uk/projects/fastqc/, accessed on 5 October 2018). Secondly, high-quality RNA-seq reads were mapped to the reference genome of *S. pombe* (NCBI RefSeq assembly: GCF_000002945.1_ASM294v2) using Hisat2 (version 2.0.5) [34] with default parameters. Thirdly, base variants were called using Samtools (version 1.7) [35] mpileup package with options “-Q 20 -ugf” and bcftools [36] with options-“vc.” The variance rate for each base variant site was estimated based on the reported numbers of reads supporting either the reference genotype or the variant genotype using Samtools (version 1.7) pileup program. The variance rate for each variant site was calculated as a percentage of reads in the variant genotype out of the total reads mapped to the site. Fourthly, base variants were filtered to identify A-to-I RNA editing events using the following criteria: (1) removing the variant sites occurring in the control strain FY7652-HFF1; (2) retaining variant sites with coverage depth ≥10; (3) retaining variant sites with a variance rate ≥10%; (4) retaining only A-to-G base-changing events. The editing levels for A-to-I RNA editing sites were estimated based on the variance rate.

### 2.7. Gene-Based Annotation of A-to-I RNA Editing Sites

A-to-I RNA editing sites were annotated using ANNOVAR (version date 8 June 2020) [37] with gene models from PomBase for *S. pombe*. These sites were annotated with gene definitions, including exonic (variants overlapping coding regions), intronic (variants overlapping introns), UTR5 (variants overlapping 5’ untranslated regions), UTR3 (variants overlapping 3’ untranslated regions), splicing (variants within 2 bp of a splicing junction), and ncRNA (variants overlapping transcripts without coding annotation in the gene definition).

Variants occurring within coding regions (CDS) are classified into two main categories: synonymous and non-synonymous. This classification is based on whether these variants impact the amino acid sequence in the resulting protein products.

### 2.8. Sequence Logo Analysis of Nucleotides Neighboring the Detected Editing Sites

The sequence logo was generated from 15 nt upstream and downstream of the A-to-I RNA editing sites identified in *S. pombe* and was analyzed using the WebLogo program [38].

### 2.9. Analysis of Minimum Free Energy for Secondary RNA Structure at Editing Sites

To compute the minimum free energy of secondary structures at A-to-I RNA editing sites, we initially extracted 200 bp sequences flanking the editing sites (100 bp upstream and 100 bp downstream). Subsequently, secondary structures were constructed for the 201 bp sequences of all sites, and the minimum free energy for each folding structure was calculated using RNAfold (version 2.6.4) from the ViennaRNA Package (version 2.6.4) [39]. We employed options ‘-T 20 -d 2 --noGU’, ‘-T 30 -d 2 --noGU’, and ‘-T 35 -d 2 --noGU’ for strains cultivated at 20 °C, 30 °C, and 35 °C, respectively.

## 3. Results

### 3.1. Construction of RNA Editing Machinery in S. pombe

**Figure 1:**
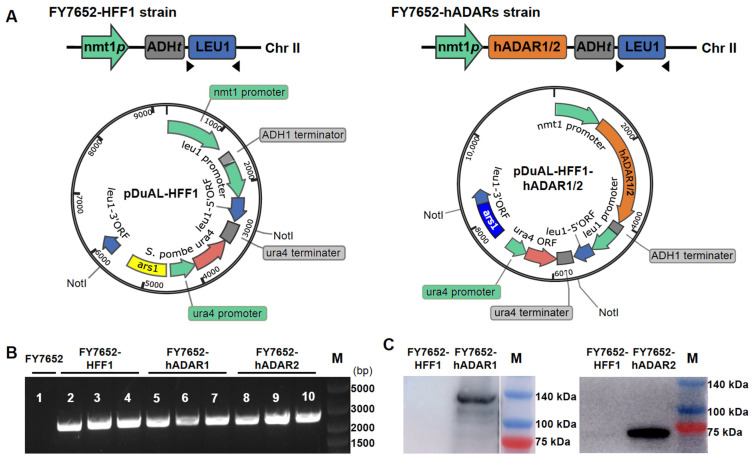
Construction of RNA editing machinery in *S. pombe* using hADAR1 or hADAR2. (**A**) Top, the schematic of the *S. pombe* chromosomal *leu1*-32 locus with or without integrated hADAR constructs. Bottom, an *E. coli—S. pombe* shuttle plasmid (pDUAL-HFF1) was modified to carry the hADARs construct. nmt1*p*, promoter; hADAR1/2, adenosine deaminases acting on RNA from humans; ADH*t*, terminator; *leu1*, gene recoding the second enzyme in leucine biosynthesis. (**B**) Confirmation of correct integration of hADAR1, hADAR2, or empty constructs into the *leu1*-32 locus on chromosome II via PCR analysis using primer sets ADHterm-F and leu1-R. Lane 1. FY7652 (no integration in LEU 1 locus); Lanes 2–4, control strain FY7652-HFF1 (*leu1*-32 locus integrated with empty construct); Lanes 5–7, FY7652-hADAR1 (*leu1*-32 locus integrated with construct of hADAR1); Lanes 8–10, FY7652-hADAR2, (*leu1*-32 locus integrated with construct of hADAR2). The target size is approximately 2.1 kb. M, DNA marker. (**C**) Expression of hADAR1 and hADAR2 detected using western blot analysis. M, protein marker.

*S. pombe* lacks the ADAR-mediated A-to-I RNA editing system, which provides a “clean” background for studying A-to-I RNA editing. To establish the RNA editing machinery in *S. pombe*, we constructed two vectors, pDUAL-HFF1-hADAR1 and pDUAL-HFF1-hADAR2 (Figure 1A, right panel), for expression of hADAR1 or hADAR2, respectively. These vectors were generated by inserting the hADAR1 and hADAR2 genes from humans into the empty vector pDUAL-HFF1 (Figure 1A, left panel). The expression of hADAR1 and hADAR2 is controlled by the *nmt1* promoter and the *ADH1* terminator.

For stable expression of hADAR1 and hADAR2 in *S. pombe*, we integrated the hADAR1 and hADAR2 constructs, along with the empty construct, into *S. pombe* chromosome II via homologous recombination, resulting in strains FY7652-hADAR1, FY7652-hADAR2, and FY7652-HFF1. The successful integrations of hADAR1, hADAR2, or the empty construct into *S. pombe* chromosome II were confirmed through PCR analysis (Figure 1B). PCR bands were observed for strains where the *leu1*-32 locus integrated with constructs, while no PCR band was detected for strains where the *leu1*-32 locus remained unaltered.

Subsequently, we tested the expression of hADAR1 and hADAR2 proteins in *S. pombe* using western blot with either anti-ADAR1 mouse monoclonal antibody or rabbit anti-ADAR2 polyclonal antibody (see Section 2 for details). Expression of hADAR1 was detected in strain FY7652-hADAR1, hADAR2 in strain FY7652-hADAR2, but not in strain FY7652-HFF1 (Figure 1C).

### 3.2. hADAR1 and hADAR2 Displaying Distinct RNA Editing Specificity in S. pombe

To explore whether the RNA editing machinery can edit RNA in *S. pombe* as it does in human cells, we cultured the RNA editing machinery containing strains FY7652-hADAR1, FY7652-hADAR2, and the control strain FY7652-HFF1 at 30 °C. Subsequently, total RNA was extracted from these cultures and subjected to transcriptome sequencing (mRNA-seq).

**Figure 2:**
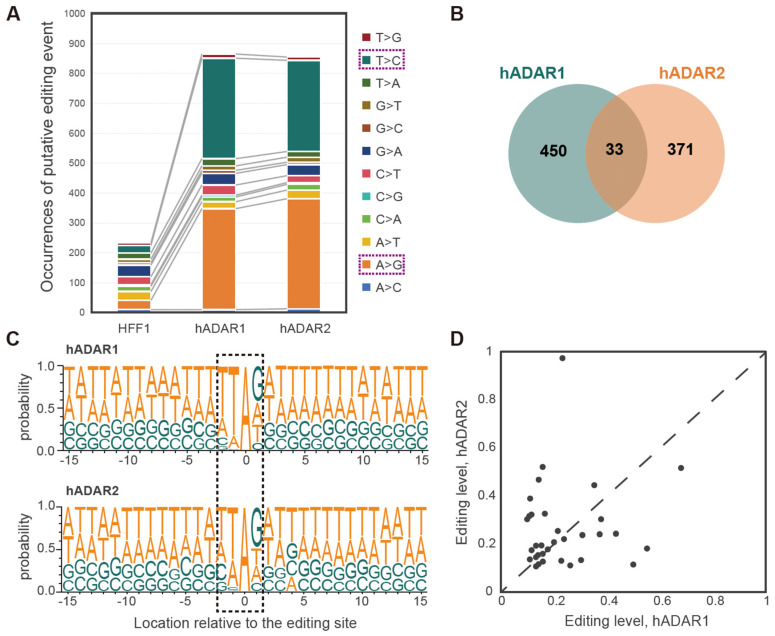
hADAR1 and hADAR2 displaying distinct RNA editing specificity in *S. pombe*. (**A**) Comparison of the RNA–DNA sequence differences pattern from strains FY7652-HFF1, FY7652-hADAR1, and FY7652-hADAR2. (**B**) Number of total and common A-to-I RNA editing sites for strains FY7652-hADAR1 and FY7652-hADAR2 illustrated using Venn diagram. (**C**) Probability sequence logo of nucleotides (+/−nucleotides) neighboring the detected editing sites between strains FY7652-hADAR1 and FY7652-hADAR2. The dashed boxes highlight the editing sites along with their adjacent nucleosides. (**D**) Comparison of A-to-I editing levels for 33 common editing sites between strains FY7652-hADAR1 and FY7652-hADAR2.

To identify and characterize A-to-I RNA editing events in strains FY7652-hADAR1, FY7652-hADAR2, we analyzed the whole deep-sequencing transcriptome data using a modified pipeline, similar to that used in our previous work [31,32] (see Section 2 for details), to call variants from strains FY7652-hADAR1, FY7652-hADAR2, and FY7652-HFF1. All 12 types of variants were identified across the three strains (Figure 2A, Supplementary Table S1). Predominantly, A-to-G and T-to-C variants constituted over 78% of total variants in strains FY7652-hADAR1 and FY7652-hADAR2. Conversely, within the control strain FY7652-HFF1, the combined proportion of A-to-G and T-to-C variants did not surpass 24% of total variants. Moreover, while the numbers of non-A-to-G and non-T-to-C variants were similar for FY7652-hADAR1, FY7652-hADAR2, and the control strain FY7652-HFF1, the number of A-to-G and T-to-C variants in strain FY7652-hADAR1 or FY7652-hADAR2 was hundreds of times greater than in the control strain FY7652-HFF1 (Figure 2A). Notably, T-to-C variants resulted from A-to-I RNA editing events occurring in transcripts that are reversely complementary to the reference genome. These data validate the RNA editing activities in *S. pombe*. By filtering out background variants from the control strain FY7652-HFF1, we identified 483 (Supplementary Table S2) and 404 (Supplementary Table S3) A to I editing sites with high confidence for strains FY7652-hADAR1 and FY7652-hADAR2, respectively. To validate the editing sites, we randomly selected 25 sites for RT-PCR and Sanger sequencing, and all of them were confirmed. (Supplementary Table S4, Supplementary Figure S1).

Given the structural similarities between the hADAR1 and hADAR2 domains, we wondered whether they exhibit similar substrate specificity. Constructing RNA editing machinery in the “clean” background of *S. pombe* provided an ideal platform for investigating this question. To address this, we compared the sets of editing sites from strains FY7652-hADAR1 and FY7652-hADAR2. Unexpectedly, we observed distinct editing profiles for strains FY7652-hADAR1 and FY7652-hADAR2, with only 33 editing sites common to both strains, accounting for 6.8% of the total editing sites in strain FY7652-hADAR1 and 8.2% in FY7652-hADAR2 (Figure 2B). This finding suggests that hADAR1 and hADAR2 have distinct editing specificities toward RNA substrates.

We then explored whether hADAR1 and hADAR2 in *S. pombe* have distinct preferences for neighboring bases, likely influencing their distinct editing specificities. Using Sample Logo sequence motifs (Figure 2C), we analyzed the neighbor preferences for the two enzymes. Both enzymes showed a stronger influence of the 5′ nearest neighbor on adenosine editing compared with the 3′ nearest neighbor, showing a preference for U and A over C and G in the 5′ nearest neighbor position. G is favored by both enzymes as the 3′ nearest neighbor, while T, A, and C are less preferred (Figure 2C). However, we observed a minor discrepancy in the neighbor preferences between hADAR1 and hADAR2. Specifically, at the −3 position relative to the editing site, hADAR1 exhibits a tendency toward T, whereas hADAR2 leans toward A. Additionally, for bases situated at positions −6 to −8 relative to the editing base A, hADAR2 tends to prefer T, while hADAR1 leans towards A. Despite the difference in the 5′ neighboring sequences for hADAR1 and hADAR2, the 3′ neighboring sequences are similar. Based on these differences in neighbor preference between hADAR1 and hADAR2, it was suggested that neighbor preference may contribute to the distinct editing profiles of hADAR1 and hADAR2 in *S. pombe*.

We further compared the editing levels for the 33 common editing sites between strains FY7652-hADAR1 and FY7652-hADAR2. We observed that while 13 sites showed a 10% higher editing level in strain FY7652-hADAR1 compared with strain FY7652-hADAR2, 14 sites exhibited a 10% higher editing level in strain FY7652-hADAR2 compared with strain FY7652-hADAR1. Additionally, six sites displayed similar editing levels in both strains (Figure 2D). Considering that hADAR1 and hADAR2 exhibited different preferences for RNA molecules related to variations in their 5′ neighboring sequences (Figure 2C), which may result in different editing activity, it indicated that the distinct editing levels observed among the 33 common editing sites stem from the different preferences of hADAR1 and hADAR2.

### 3.3. Varying Temperature Affecting RNA Editing by hADAR1 and hADAR2 in S. pombe

**Figure 3:**
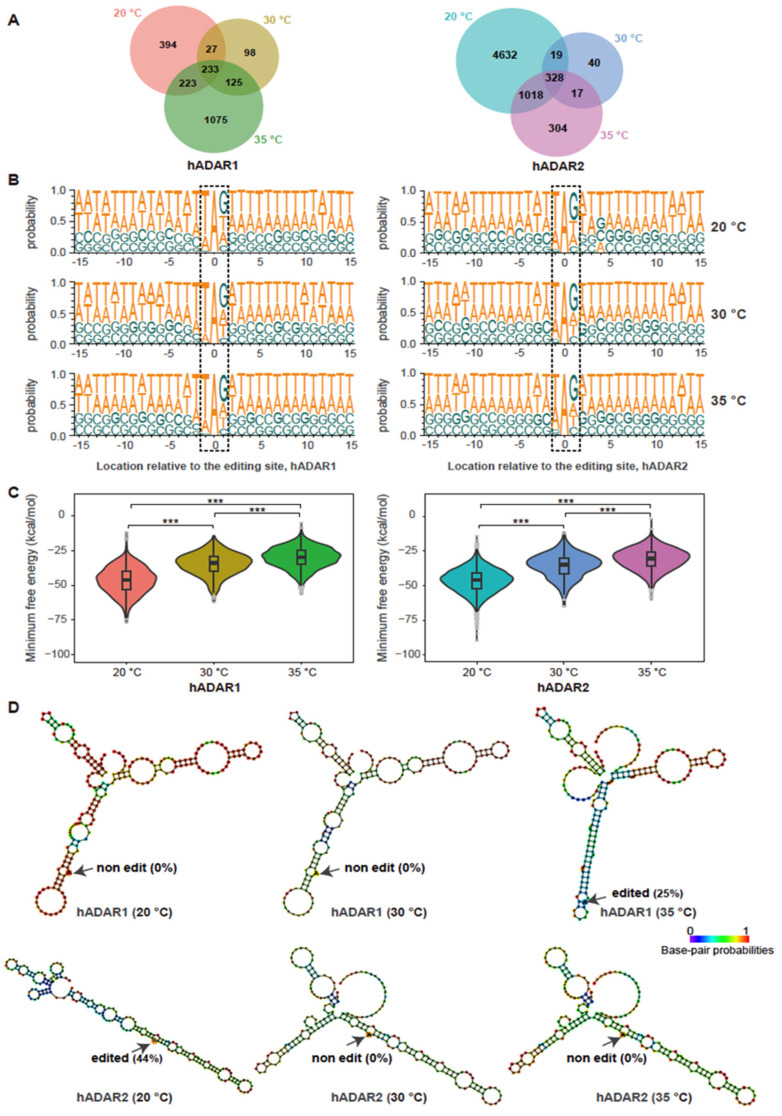
Comparison of A-to-I editing specificity for hADAR1 and hADAR2 in *S. pombe* at different temperatures. (**A**) Number of total and common A-to-I RNA editing sites for strains FY7652-hADAR1 and FY7652-hADAR2 across different temperatures, illustrated using Venn diagram. (**B**) Probability sequence logo of nucleotides neighboring the detected editing sites in strains FY7652-hADAR1 and FY7652-hADAR2 across different temperatures. The dashed boxes highlight the editing sites along with their adjacent nucleosides. (**C**) Comparison of the minimum free energy of secondary structures surrounding RNA editing sites across various temperatures. The RNA structures were determined using RNAfold, and the minimum free energy was calculated accordingly. Mann-Whitney rank test was performed to obtain the *p* value (***, *p* < 0.001). (**D**) Examples of the in silico prediction of secondary structures surrounding two RNA editing sites at three temperatures using RNAfold. Top panel, RNA adopts a structure favorable for hADAR1 editing at 35 °C (right in top panel), achieving a 25% editing efficiency, while no editing occurred at 20 °C (left in top panel) or 30 °C (middle in top panel) with corresponding RNA structures. Bottom panel: RNA adopts a structure suitable for hADAR2 editing at 20 °C (left in top panel), resulting in editing with 44% efficiency, whereas no editing was observed at 30 °C (middle in bottom panel) and 35 °C (right in bottom panel) with corresponding RNA structures.

To investigate the effect of temperature on editing, we conducted a comparative analysis of RNA editing events for strains FY7652-hADAR1 or FY7652-hADAR2 across three different temperatures: 20 °C, 30 °C, and 35 °C. For strain FY7652-hADAR1, 877, 483, and 1656 editing sites were identified at 20 °C, 30 °C, and 35 °C, respectively (Figure 3A). Conversely, FY7652-hADAR2 had 5997, 404, and 1667 editing sites at the same respective temperatures. Notably, distinct sets of editing sites were observed for both FY7652-hADAR1 and FY7652-hADAR2 across the different temperature conditions, with a higher number of editing sites observed at 20 °C and 35 °C compared with 30 °C. Previous studies have indicated that ADAR enzymes from ectothermic organisms such as *Drosophila* and octopuses can edit RNA at different sites depending on temperature, thereby facilitating adaptation to diverse thermal environments [40,41]. Our finding suggested that hADARs derived from endothermic animals like humans retained the capacity to edit RNA at distinct sites in response to variant temperatures. In strain FY7652-hADAR1, 233 editing sites were common across all temperatures, representing 26.5%, 48.2%, and 14.1% of the total editing sites at 20 °C, 30 °C, and 35 °C, respectively. Similarly, FY7652-hADAR2 shared 328 editing sites across all temperatures, accounting for 5.5%, 81.2%, and 19.7% of the total editing sites at 20 °C, 30 °C, and 35 °C, respectively. Compared with the ratio of shared editing sites between hADAR1 and hADAR2 to the total editing sites of either hADAR1 (6.8%, Figure 2B) or hADAR2 (8.2%, Figure 2B), the ratio of shared editing sites for a specific enzyme across different temperatures to the total editing sites at either 20 °C, 30 °C, or 35 °C is larger. This suggests that the type of enzyme, whether hADAR1 or hADAR2, has a greater influence on RNA editing specificity than temperature does within the temperature range of 20 °C to 35 °C.

We further explored whether the hADARs enzyme in *S. pombe* has changed preference for neighboring bases across different temperatures, which may potentially result in their distinct editing sites across different temperatures. Employing Sample Logo sequence motifs (Figure 3B), we analyzed the neighboring preferences for the two enzymes across various temperatures. Interestingly, we found that both hADAR1 and hADAR2 maintain consistent 5′ and 3′ nearest neighbor preferences across three different temperatures, with U and G being the most preferred 5′ and 3′ nearest neighbors, respectively. However, we observed a minor discrepancy in the 5’ nearest neighbor preferences between hADAR1 and hADAR2, with hADAR2 showing a stronger preference for A (Supplementary Figure S2). Additionally, some subtle differences were noted in the probability sequence logo of Figure 3B for neighbors situated at +2 to +15 bases and −2 to −15 bases away from the editing site for both hADAR1 and hADAR2 across three different temperatures; there were no significant shifts observed across the temperature range from 20 °C to 35 °C. This finding suggested that the sequence preferences for neighboring bases of editing sites for both hADAR1 and hADAR2 were unaffected by temperature variations in the range of 20 °C to 35 °C.

The secondary structure of dsRNA is influenced by ambient temperature [40,42]. Previous studies have indicated that RNA editing relies on the recognition and binding of dsRNA secondary structures by the dsRNA binding domains (dsRBDs) [16]. We then wondered whether the stability of RNA structures across different temperatures contributes to the difference in editing sites observed across various temperatures for both enzymes. To explore this possibility, we performed in silico analysis for RNA structures surrounding temperature-specific editing sites using RNAfold. We calculated the minimum free energy for these secondary structures. As expected, the minimum free energy for RNA substrates of both hADAR1 and hADAR2 decreased with temperature, indicating more stable RNA structures at lower temperatures (Figure 3C). We did not observe any correlation between the minimum free energy and the number of editing sites at different temperatures for both hADAR1 and hADAR2. It is possible that factors such as the length of the double-stranded RNA where the editing site is located, as well as the presence and position of loops and bubbles within the secondary structure, all influence the occurrence of RNA editing. For instance, at 35 °C, a longer length of dsRNA surrounding the editing site, compared with that at 20 °C and 30 °C, facilitated A-to-I editing by hADAR1, while no RNA editing occurred at 20 °C and 30 °C, despite the higher minimum free energy for RNA structures at 35 °C than at 20 °C and 30 °C (Figure 3D, top panel). Another example of A-to-I editing across three different temperatures showed that at 20 °C, the targeted base A was situated within a more stable dsRNA structure, resulting in an editing level of 44% by hADAR2 (Figure 3D, bottom panel). No RNA editing occurred at this site when the temperature was either 30 °C or 35 °C.

We then wonder whether the expression levels of the two ADAR enzymes across three temperatures contribute to the variation in editing sites observed at different temperatures. To explore this, we initially quantified the expression levels of hADAR1 and hADAR2 under three temperatures using the metric of transcripts per million reads (TPM). For hADAR1, the TPM values were 2480.08 at 20 °C, 3897.72 at 30 °C, and 6295.82 at 35 °C, indicating a positive correlation between temperature and transcript levels. Conversely, for hADAR2, the TPM values were 8516.75 at 20 °C, 7005.32 at 30 °C, and 10,306.94 at 35 °C, suggesting that hADAR2 exhibits no significant variations with temperature changes. Subsequently, we examined the relationship between the expression of hADAR enzymes and the number of editing sites at various temperatures. Similar trends in response to temperature were observed for hADAR2 enzyme expression and RNA editing site frequency. At 30 °C, both hADAR2 enzyme expression and RNA editing site numbers reach their lowest point. Interestingly, a decrease to 20 °C or an increase to 35 °C is accompanied by an increase in hADAR2 expression and a rise in editing sites. For hADAR1, a correlation was evident at 30 °C and 35 °C, with an increased number of editing sites correlating with hADAR1 transcription level. Interestingly, at 20 °C, the number of editing sites increased despite a decrease in hADAR1 transcription. This unexpected increase in editing sites at lower temperatures may be due to the enhanced stability of RNA structures, which could provide more targets for editing by hADAR1.

### 3.4. Genes’ Functions Possibly Affected by the Editing of Their Transcript

**Figure 4:**
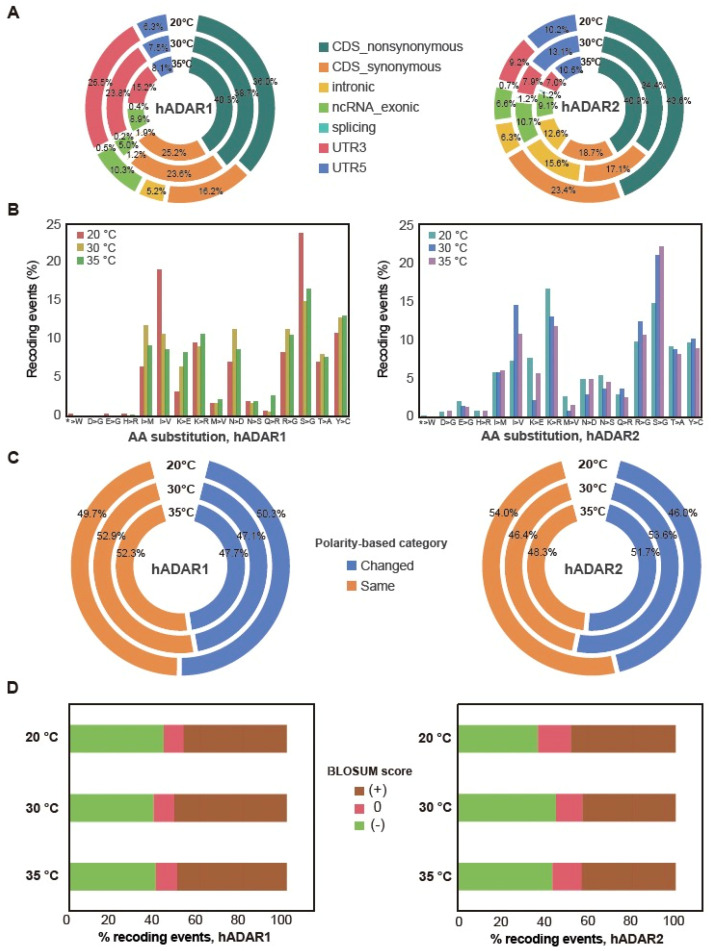
Amino-acid substitutions due to A-to-I RNA editing by hADAR1 or hADAR2 and their related function. (**A**) The genome distribution of A-to-I editing events in *S.pombe* strains induced by hADARs at different temperatures. CDS_nonsynonymous, the proportion of non-synonymous amino acid; CDS_synonymous, the proportion of synonymous amino acid. (**B**) Frequencies of amino acid substitutions due to A-to-I RNA editing by hADAR1 or hADAR2 in *S. pombe* across three temperatures. In the *x*-axis, the symbol “*” signifies the presence of the stop codons TAG and TGA. (**C**) The proportion of recoding sites where amino acid substitutions remain within the same polarity category across different temperatures induced by hADAR1 or hADAR2. (**D**) The fraction of recoding sites resulting in evolutionarily common amino acid substitutions (positive BLOSUM scores) across different temperatures by hADAR1 or hADAR2.

We next assessed the potential functional impacts of A-to-I RNA editing on mRNAs. We used ANNOVAR to annotate the effects of A-to-I editing on transcripts based on their gene annotations (Section 2) by either hADAR1 or hADAR2. We found that RNA editing sites reside across all six types of gene structures, including 5′-UTR, 3′-UTR, splicing regions, non-coding RNAs (ncRNA), introns, and exons (Figure 4A). Interestingly, over 50% of the editing sites reside in CDS in both strains, FY7652-hADAR1 and FY7652-hADAR2. Notably, for FY7652-hADAR1, the 3′-UTR region emerged as the second-most populated area for editing sites, accounting for 25.5%, 23.8%, and 15.2% of the total editing sites at different temperatures, while for FY7652-hADAR2, the intron region held the second-highest proportion of editing sites, accounting for 15.6%, 12.6%, and 6.3% of the total editing sites at different temperatures. This difference underscores the distinct editing specificity between hADAR1 and hADAR2.

Editing sites within exons possess the potential to induce synonymous or non-synonymous recoding, start-loss, or stop-loss mutations. Given that over 50% of the editing sites are located within CDS in both strains, we are intrigued by the extent to which these sites could induce recoding. We found that over 60% of editing sites within the exon region were non-synonymous, resulting in amino acid changes in the protein for both the two strains across three temperatures (Figure 4B). Start-loss and stop-loss mutations were also found for editing sites within exons in the two strains.

Non-synonymous recoding resulting from A-to-I editing can alter amino acids across sixteen types. In both strains, FY7652-hADAR1 and FY7652-hADAR2, the most prevalent recoding events are the substitution from serine to glycine (S > G) (Figure 4B). Upon reanalyzing the sequences of transcripts containing these S > G recoding events, we observed that the codons for serine are AGU and AGC, with the first base A in the two codons being edited. This highlights the preference of G for the 3’ nearest neighbor of the editing site A. Furthermore, for the AGU and AGC codons of serine with A being edited, the 5’ nearest base is predominantly T, with a ratio of 84.2% (s.e.m. = 3.8%), followed by A (11.3%, s.e.m. = 3.1%) or C (4.2%, s.e.m. = 1.1%). This pattern reflects the preference of U for the 5’ nearest neighbor of the editing site A. This finding further confirms that hADAR enzymes preferentially edit A with a 5′ nearest base of T and a 3′ nearest base of G. We also observed different ratios for specific conversions between strains FY7652-hADAR1 and FY7652-hADAR2. For example, strain FY7652-hADAR2 exhibited more lysine to arginine substitution (K > R) compared with that of FY7652-hADAR1, whereas strain FY7652-hADAR1 displayed a higher propensity for tyrosine to cysteine substitution (Y > C) conversion than strain FY7652-hADAR2. These discrepancies in amino acid substitution patterns between FY7652-hADAR1 and FY7652-hADAR2 underscore the distinct editing sites of hADAR1 and hADAR2.

When comparing the amino acid substitution patterns between different temperatures, we observed similarities between patterns at 30 °C and 35 °C for both strains FY7652-hADAR1 and FY7652-hADAR2. However, distinct amino acid substitution patterns were observed at 20 °C compared with both 30 °C and 35 °C. At 20 °C, strain FY7652-hADAR1 exhibited higher proportions of S > G and I > V substitutions, along with fewer K > R substitutions, in contrast to its patterns at 30 °C and 35 °C. Conversely, strain FY7652-hADAR2 exhibited the opposite trend: lower proportions of S > G and I > V substitutions and higher rates of K > E and K > R conversions at 20 °C compared with its patterns at higher temperatures.

This discrepancy prompted us to investigate whether these different amino acid substitution patterns lead to varied changes in amino acid polarity, potentially indicating involvement in temperature adaptation. We performed amino acid polarity analysis for the recoding events, categorizing them as “Change” or “Same” based on alterations in amino acid polarity (Supplementary Table S5). We found that, across all three temperatures, the proportion of editing events for hADAR1, maintaining the polarity of amino acids as “Same” remained consistent. For hADAR2, the proportion of editing events maintaining the polarity of amino acids as “Same” is slightly higher at 20 °C compared with that at 30 °C and 35 °C (Figure 4C, Supplementary Table S5). In addition to the proportion of editing events, maintaining the polarity of amino acids as “Same” and “Change” is comparable for both hADAR1 and hADAR2. This revealed that while hADAR1 maintains a consistent polarity in amino acid substitutions across different temperatures, hADAR2 shows a slight increase in maintaining the same polarity at lower temperatures, suggesting a potential role for hADAR2 in low-temperature adaptation.

We further analyzed the BLOSUM (blocks substitution matrix 80) score for the editing-induced amino acid substitutions. Positive BLOSUM80 scores indicate evolutionarily conserved amino acid substitutions, whereas negative scores indicate evolutionarily un-conserved substitutions. We found that the ratio of evolutionarily conserved substitutions is comparable to that of un-conserved substitutions for strains FY7652-hADAR1 and FY7652-hADAR2 across three different temperatures (Figure 4D). This result reflects the fact that these RNA editing sites are random in *S. pombe* and do not happen naturally because of the absence of ADAR enzymes.

## 4. Discussion

Adenosine-to-inosine (A-to-I) RNA editing is a conserved post-transcriptional mechanism in metazoans catalyzed by ADAR enzymes. This process generates diverse transcript isoforms and can influence various biological processes, including protein recoding, alternative splicing, and microRNA-mediated gene regulation, in response to varying conditions. While RNA editing is generally functional or adaptive in metazoans, it is absent in bacteria, fungi, and plants, where the three ADAR enzymes are not present. In *S. pombe*, although an ADAR-like deaminase domain containing protein TAD1 is known to convert adenosine at position 37 of tRNA^Ala^ to inosine [43], until now, no ADARs responsible for RNA A-to-I editing in mRNA have been detected in *S. pombe*. We introduced the A-to-I RNA editing system into fission yeast *S. pombe* and identified hundreds to thousands of A-to-I editing sites for both enzymes across different temperatures through RNA-seq data analysis. Despite the lack of replication for RNA-seq data, we have utilized Sanger sequencing to validate the RNA A-to-I editing sites identified through RNA-seq. Notably, the number of editing sites identified in *S. pombe* in this study surpasses that reported in *Schizosaccharomyces cerevisiae* containing hADAR1 or hADAR2 by over one hundredfold [16,17]. This abundance of editing sites in *S. pombe* provides an exceptional platform for studying ADAR enzyme specificity.

Despite sharing a structural similarity, hADAR1 and hADAR2 enzymes exhibit preferences for targeting different editing sites, with only a small overlap in their editing repertoire in *S. pombe* (Figure 2A). A similar pattern was observed for hADAR1 and hADAR2 in human cells [14], whereas mice exhibited a more extensive shared editing site profile for ADAR1 and ADAR2. This specificity could be elucidated by their different preferences for neighboring sequences, particularly the disparities in their 5′ neighboring sequences (Figure 2D). In *S. cerevisiae*, the sequence preference analysis of hADAR1 and hADAR2 did not reveal significant differences, possibly due to limited data availability [13,17]. However, in mice, a nuanced preference for neighboring bases was noted; mouse ADAR1 prefers thymine at the -2 position relative to the target adenosine, while ADAR2 favors cytosine at the same position [15]. These neighboring nucleotides reflect the ability of the target adenosine to flip out of the duplex and undergo deamination [44]. The 5′ side of the editing site interacts with a protein loop in hADAR2d, as revealed by the crystal structures of hADAR2d bound to RNA [45]. It has been hypothesized that a 5′-G would hinder the hADAR2 protein at G489, whereas a U or A would not encounter this hindrance [46]. Swapping the protein loop (aa972-aa99) in hADAR1 with that (aa457-aa479) from hADAR2 shifts the substrate preference of hADAR1 to that of hADAR2 [17]. The deaminase domains of hADAR1 and hADAR2 have relatively low sequence homology in their 5′ binding loops compared with other regions, suggesting one possible reason for the difference in their substrate selectivity [46]. This was consistent with our finding that the preferences for 5′ neighboring sequences differ between the two enzymes hADAR1 and hADAR2, while the preferences for the 3′ neighboring sequences are comparative (Figure 2D). The preference for a G at the 3′ position is hypothesized due to hydrogen bonding between the 2-amino group of G and the backbone carbonyl group of S486 in the 3′ neighboring sequences’ binding loops. The 3′ neighboring sequences’ binding loops are highly similar for ADAR1 and ADAR2 [47]. It was believed that the preferences for the 3′ neighboring sequences are related to the dsRBM of the enzyme [48].

Our study reveals that hADAR1 and hADAR2 derived from endothermic humans possess the capacity to edit distinct RNA sites in response to environmental temperature changes when expressed in *S. pombe* (Figure 3A). In ectotherms, A-to-I RNA editing can respond to temperature changes, resulting in different RNA editing events. In flies, decreased temperatures lead to increased editing at 55 additional protein coding (CDS) sites [40]. In octopuses, 20,850 editing sites were identified as cold-induced, while only 789 editing sites were warm-induced [41]. Furthermore, in hibernating ground squirrels, 5165 A-to-I editing sites exhibited a dynamic increase in editing level after prolonged cold exposure [49]. However, in *S. pombe*, although the number of editing sites for both hADAR1 and hADAR2 increased at 20 °C compared with 30 °C, the number of editing sites at 35 °C did not decrease compared with that at 30 °C. There is no correlation between the number of editing sites and the temperature across the range of 20 °C to 35 °C. This suggested that ADARs from endotherms retain the ability to respond to temperature changes and edit different sets of editing sites. However, the number of editing sites did not systematically increase as temperatures decreased from 35 °C to 30 °C and then to 20 °C. RNA editing at different temperatures can be affected by factors such as RNA structural stability, dsRNA length at editing sites, and secondary structure features like loops and bulges, impacting ADAR enzyme access to these editing sites. Additionally, variations in the transcriptome at different temperatures may also impact RNA editing. Changes in gene expression levels could alter the availability of substrates for ADAR enzymes, thus influencing the overall editing activity.

In *S. pombe*, more than 50% of editing sites caused by hADAR1 and hADAR2 are found in the coding sequence (Figure 4A), a pattern notably distinct from observations in mammals. In mice, 85% of editing sites occur within repeat elements, whereas in humans, 98% of editing sites are located within repeat elements [50]. In hibernating ground squirrels, 99.6% of cold-induced editing sites are located outside the coding sequence [49]. These distributional differences in RNA editing sites across genomes likely stem from variations in genomic architecture, including the proportion of exons, introns, and intergenic regions. In humans, these components account for 1.1%, 24%, and 75% of the genome, respectively [51]. In contrast, in mice, exons represent a mere 1–2% of the entire genome [52]. *S. pombe* exhibits a distinct pattern of exons, introns, and intergenic regions comprising 60.2%, 3.2%, and 39.8% of the genome respectively [53]. Moreover, in *S. pombe*, over 60% of editing sites within the coding sequence were non-synonymous, closely resembling observations in Coleoid cephalopods, which exhibit an abundance of non-synonymous RNA editing sites [54]. In Cephalopoda, A-to-I RNA editing plays a functional role in response to temperature fluctuations. For example, RNA editing and recoding tunes protein functions such as K+ channel Kv1.1 [55], synaptotagmin, kinesin-1 [41], and microtubule motor protein [56], enabling them to acclimate to cold temperatures. It has been reported that in octopuses, cold-induced editing events not only tend to preserve the polarity of the amino acids, potentially aiding in acclimation to cold temperatures [41], but also result in a significantly higher rate of conserved amino acid substitutions under varying temperatures. In contrast, in yeast, the ratio of recoding events that maintain the polarity of amino acids and the ratio that preserves amino acid conservation show no significant differences across different temperatures. This may be due to the fact that RNA editing sites in yeast are naturally occurring. In addition, evolutionary analysis suggests that conserved non-synonymous editing sites with high editing levels in *Drosophila* and Coleoid cephalopods may be adaptive [57]. Thus, whether the presence of numerous non-synonymous RNA editing sites in fission yeast can serve any functional purpose or contribute to the adaptability of fission yeast to various environments post-domestication has not been explored in this study and warrants further research in the future.

In summary, we established the RNA editing machinery in *S. pombe* and observed distinct editing specificity between the two human ADAR enzymes hADAR1 and hADAR2, which may stem from different neighboring preferences. Furthermore, we observed that both hADAR1 and hADAR2 exhibited altered RNA editing specificity under different temperature conditions, while their preference for the neighboring sequences remained unchanged. Additionally, our findings demonstrate that A-to-I RNA editing extensively recodes proteins in fission yeast. This study revealed the extensive differences in substrate selectivity between the two active human ADARs, i.e., ADAR1 and ADAR2, and provided novel insight when utilizing the two different enzymes for in vivo treatment of human genetic diseases using the RNA editing approach.

Our study also has several limitations. Firstly, we utilize *S. pombe* as a tool to investigate the specificity of hADARs, the impact of temperature on editing, and the changes in gene function due to editing. However, there are significant differences between fission yeast and human cells, such as base composition, genome organization, cellular environment, life cycle, and evolutionary status. These differences may affect the outcomes of RNA editing, potentially reducing the direct comparability of results obtained from yeast to those from humans. Secondly, while ADAR enzymes are inherently present in human cells, the RNA editing sites in human cells may be the result of evolutionary selection. In contrast, fission yeast does not inherently possess ADAR enzymes, suggesting that any RNA editing sites in yeast are random and have not undergone evolutionary selection. Nonetheless, these sites are still subject to specific recognition by ADAR enzymes, albeit without the influence of evolutionary pressures.

## Data Availability

The raw data supporting the conclusions of this article will be made available by the authors on request.

## Supplementary Materials

The following supporting information can be downloaded at: https://www.mdpi.com/article/10.3390/genes15070898/s1, Figure S1: Confirmation of candidate A-to-I RNA editing sites by sanger sequencing. The purple-dash boxes denote the detected editing event. Figure S2: Bar plot showing the proportion of T or A at the 5′ nearest neighbor of the adenosine editing site. The *p* values from χ^2^ tests are shown above each comparison. Table S1: Statistics of RNA–DNA sequence variants identified from strains FY7652-HFF1, FY7652-hADAR1, and FY7652-hADAR2. Table S2: Editing sites identified from strain FY7652-hADAR1. Table S3: Editing sites identified from strain FY7652-hADAR2. Table S4: Twenty-five RNA A-to-I editing sites of hADAR2 verified by Sanger sequencing. Table S5: Statistics of amino acid substitution and polarity-based categories in the recoding events in strains FY7652-hADAR1 and FY7652-hADAR2.
