# Supplementary material for: Revealing Differential RNA Editing Specificity of Human ADAR1 and ADAR2 in Schizosaccharomyces pombe
Source: Genes (Basel). 2024 Jul 9;15(7):898. doi: 10.3390/genes15070898 (PMC11276115; doi:10.3390/genes15070898)
Supplement: Supplementary file 1 [file genes-15-00898-s001.zip › Supplementary Figure S1.pdf]

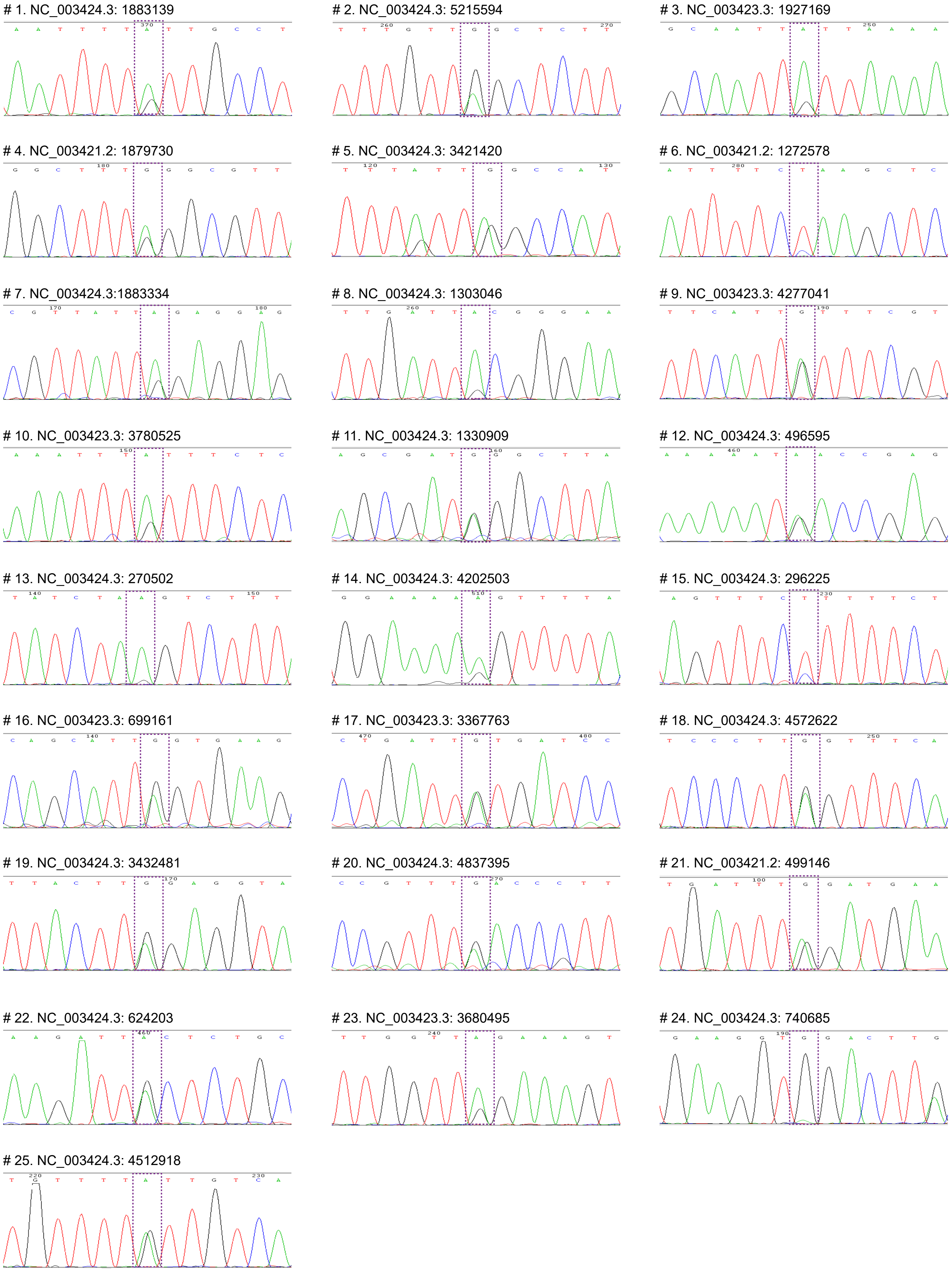


Supplementary Figure S1. Confirmation of candidate A-to-I RNA editing sites by sanger sequencing. The purple dashed boxes denote the detected editing events.
