# Supplementary material for: Revealing Differential RNA Editing Specificity of Human ADAR1 and ADAR2 in Schizosaccharomyces pombe
Source: Genes (Basel). 2024 Jul 9;15(7):898. doi: 10.3390/genes15070898 (PMC11276115; doi:10.3390/genes15070898)
Supplement: Supplementary file 1 [file genes-15-00898-s001.zip › Supplementary Figure S2.pdf]

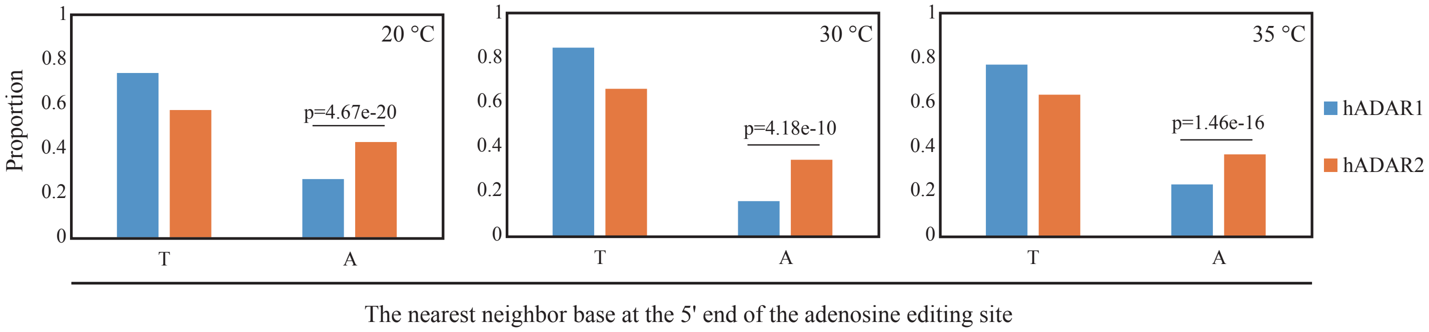

Supplementary Figure S2. Bar plot showing the proportion of T or A at the 5' nearest neighbor of the adenosine editing site. The p values from χ^2 tests are shown above each comparison.
