# Supplementary material for: Revealing Differential RNA Editing Specificity of Human ADAR1 and ADAR2 in Schizosaccharomyces pombe
Source: Genes (Basel). 2024 Jul 9;15(7):898. doi: 10.3390/genes15070898 (PMC11276115; doi:10.3390/genes15070898)
Supplement: Supplementary file 1 [file genes-15-00898-s001.zip › Supplementary Table S4. Twenty-five RNA A-to-I editing sites of hADAR2 verified by Sanger sequencing.pdf]

Supplementary Table S4. Twenty-five RNA A-to-I editing sites of hADAR2 verified by sanger sequencing.

# No.	Gene name	Genomic location	Sequence (5' – 3')	EditRatio	Editing condition*
# 1	SPAPB1A10.11c	NC_003424.3: 1883139	5'-TCTTAATTTTATTGCCTTAAT-3'	0.14	20℃
# 2	SPAC19D5.04	NC_003424.3: 5215594	5'-CGTTTTTGTTAGCTCTTCCGG-3'	0.15	20℃
# 3	SPBC16E9.07	NC_003423.3: 1927169	5'-TTCAGCAATTATTAAAAGAAT-3'	0.15	20℃
# 4	SPCC297.03	NC_003421.2: 1879730	5'-TATGGGCTTTAGGCGTTACTT-3'	0.15	20℃
# 5	SPAC3H5.09c	NC_003424.3: 3421420	5'-TCTTTTTATTAGCCATATGTA-3'	0.16	20℃
# 6	SPCC23B6.03c	NC_003421.2: 1272578	5'-CCAAGAGCTTAGAAAATTTTT-3'	0.19	20℃
# 7	SPAPB1A10.11c	NC_003424.3:1883334	5'-TCACGTTATTAGAGGAGAGGA-3'	0.2	20℃
# 8	SPAC1565.07c	NC_003424.3: 1303046	5'-CAGCTTGATTACGGGAAGCTA-3'	0.21	20℃
# 9	SPBC16A3.11	NC_003423.3: 4277041	5'-ACAGTTCATTATTTCGTGGCA-3'	0.21	20℃
# 10	SPBC31F10.14c	NC_003423.3: 3780525	5'-AGCAAAATTTATTTCTCAATT-3'	0.25	20℃
# 11	SPAC6F12.10c	NC_003424.3: 1330909	5'-TGCAAGCGATAGGCTTAGAAC-3'	0.28	20℃
# 12	SPAPB21F2.02	NC_003424.3: 496595	5'-TTCCAAAAATAACCGAGCTTT-3'	0.28	20℃
# 13	SPAC1F5.11c	NC_003424.3: 270502	5'-GCAGTATCTAAGTCTTTATGT-3'	0.31	20℃

# 14	SPAC694.02	NC_003424.3: 4202503	5'-CGATGGAAAAAGTTTTACGTG-3'	0.41	20℃
# 15	SPAC18B11.11	NC_003424.3: 296225	5'-GCTTAGAAAAAGAAACTATTC-3'	0.31	20℃ | 35℃
# 16	SPBPJ4664.02	NC_003423.3: 699161	5'-TAGCAGCATTAGTGAAGCGTC-3'	0.35	20℃ | 35℃
# 17	SPBC11C11.11c	NC_003423.3: 3367763	5'-AACCCTGATTATGATCCTAAA-3'	0.42	20℃ | 35℃
# 18	SPAC12B10.01c	NC_003424.3: 4572622	5'-ATGGTCCCTTAGTTTCACTTA-3'	0.78	20℃ | 35℃
# 19	SPAC3H5.06c	NC_003424.3: 3432481	5'-CTGATTACTTAGAGGTAATTT-3'	0.35	20℃ | 30℃ | 35℃
# 20	SPAC4F10.04	NC_003424.3: 4837395	5'-CTTTCCGTTTAACCCTTTAGT-3'	0.36	20℃ | 30℃ | 35℃
# 21	SPCC970.08	NC_003421.2: 499146	5'-AGCATGATTTAGATGAAGTTA-3'	0.48	20℃ | 30℃ | 35℃
# 22	SPAC1F3.05	NC_003424.3: 624203	5'-ACTGAAGATTACTCTGCCTTG-3'	0.79	20℃ | 30℃ | 35℃
# 23	SPBP8B7.22	NC_003423.3: 3680495	5'-GTATTTGGTTAGAAAGTGTTG-3'	0.79	20℃ | 30℃ | 35℃
# 24	SPAC22G7.06c	NC_003424.3: 740685	5'-TAATGAAGGTAGACTTACTTT-3'	0.84	20℃ | 30℃ | 35℃
# 25	SPAC27D7.03c	NC_003424.3: 4512918	5'-GTAATGTTTTATTGTCAGCTG-3'	0.86	20℃ | 30℃ | 35℃

* 20℃: the verified A-to-I RNA editing sites only detected in FY7652-hADAR2 cultured at 20℃; 20℃ | 35℃: the verified A-to-I RNA editing sites detected in FY7652-hADAR2 cultured at 20℃ and 35℃; 20℃ | 30℃ | 35℃: the verified A-to-I RNA editing sites detected in FY7652-hADAR2 cultured at 20℃, 30℃ and 35℃.
